# Collection of Viable Aerosolized Influenza Virus and Other Respiratory Viruses in a Student Health Care Center through Water-Based Condensation Growth

**DOI:** 10.1128/mSphere.00251-17

**Published:** 2017-10-11

**Authors:** Maohua Pan, Tania S. Bonny, Julia Loeb, Xiao Jiang, John A. Lednicky, Arantzazu Eiguren-Fernandez, Susanne Hering, Z. Hugh Fan, Chang-Yu Wu

**Affiliations:** a Department of Environmental Engineering Sciences, Engineering School of Sustainable Infrastructure and Environment, University of Florida, Gainesville, Florida, USA; b Department of Environmental and Global Health, College of Public Health and Health Professions, University of Florida, Gainesville, Florida, USA; c Emerging Pathogens Institute, University of Florida, Gainesville, Florida, USA; d J. Crayton Pruitt Family Department of Biomedical Engineering, University of Florida, Gainesville, Florida, USA; e Aerosol Dynamics Inc., Berkeley, California, USA; f Department of Mechanical and Aerospace Engineering, University of Florida, Gainesville, Florida, USA; University of Michigan—Ann Arbor

**Keywords:** infectious agent, infirmary, sampling, transmission, virus aerosol

## Abstract

The dynamics and significance of aerosol transmission of respiratory viruses are still controversial, for the major reasons that virus aerosols are inefficiently collected by commonly used air samplers and that the collected viruses are inactivated by the collection method. Without knowledge of virus viability, infection risk analyses lack accuracy. This pilot study was performed to (i) determine whether infectious (viable) respiratory viruses in aerosols could be collected from air in a real world environment by the *vi*able *v*irus *a*erosol *s*ampler (VIVAS), (ii) compare and contrast the efficacy of the standard bioaerosol sampler, the BioSampler, with that of the VIVAS for the collection of airborne viruses in a real world environment, and (iii) gain insights for the use of the VIVAS for respiratory virus sampling. The VIVAS operates via a water vapor condensation process to enlarge aerosolized virus particles to facilitate their capture. A variety of viable human respiratory viruses, including influenza A H1N1 and H3N2 viruses and influenza B viruses, were collected by the VIVAS located at least 2 m from seated patients, during a late-onset 2016 influenza virus outbreak. Whereas the BioSampler when operated following our optimized parameters also collected virus aerosols, it was nevertheless overall less successful based on a lower frequency of virus isolation in most cases. This side-by-side comparison highlights some limitations of past studies based on impingement-based sampling, which may have generated false-negative results due to either poor collection efficiency and/or virus inactivation due to the collection process.

**IMPORTANCE** The significance of virus aerosols in the natural transmission of respiratory diseases has been a contentious issue, primarily because it is difficult to collect or sample virus aerosols using currently available air sampling devices. We tested a new air sampler based on water vapor condensation for efficient sampling of viable airborne respiratory viruses in a student health care center as a model of a real world environment. The new sampler outperformed the industry standard device (the SKC BioSampler) in the collection of natural virus aerosols and in maintaining virus viability. These results using the VIVAS indicate that respiratory virus aerosols are more prevalent and potentially pose a greater inhalation biohazard than previously thought. The VIVAS thus appears to be a useful apparatus for microbiology air quality tests related to the detection of viable airborne viruses.

## Abstract (precis)

The significance of virus aerosols in the natural transmission of respiratory diseases has been a contentious issue, primarily because it is difficult to collect or sample virus aerosols using currently available air sampling devices. We tested a new air sampler based on water vapor condensation for efficient sampling of viable airborne respiratory viruses in a student health care center as a model of a real world environment. The new sampler outperformed the industry standard device (the SKC BioSampler) in the collection of natural virus aerosols and in maintaining virus viability. These results using the VIVAS indicate that respiratory virus aerosols are more prevalent and potentially pose a greater inhalation biohazard than previously thought. The VIVAS thus appears to be a useful apparatus for microbiology air quality tests related to the detection of viable airborne viruses.

## INTRODUCTION

Airborne infectious agents in health care facilities pose risks to both patients and employees. Nosocomial transmission of human influenza virus and other respiratory viruses is a major concern in health care facilities, especially because immunocompromised patients who may be present in those settings are at greater risk of getting infected and are more vulnerable to the development of severe disease. Transmission of influenza viruses from one person to another can occur by three routes: direct contact of infectious secretions with mucus membranes of the upper respiratory tract (URT), contact of virus-containing large droplet sprays with surfaces of the URT, and inhalation of small aerosols and droplet nuclei that then deposit in the lower respiratory tract (LRT) (1–3) A fourth route has been shown in animal models: ocular infection, wherein airborne influenza viruses come into contact with ocular surfaces (4). The relative importance of each transmission route is poorly understood and probably varies depending on virus strain, environmental conditions, etc. (3). Among them, the aerosol transmission mode is the most contentious one with regard to (i) economic reasons, as expensive precautions would be needed for implementation of appropriate infection control processes in health care settings and (ii) the fact that no clear evidence exists from which one can extrapolate results from lab experiments to real life and from animals to human beings (5), as the artificial aerosols produced in a lab are quite different from natural aerosols generated by coughing, sneezing, and other activities. Unfortunately, from an infection control perspective, if people can get infected via aerosol transmission, additional interventions, such as the use of N95 respirators rather than surgical masks, increased air ventilation, isolation of infected patients, and use of a filtration system used for large droplets would be advised, and this would increase health care costs. Therefore, new sampling methods are needed to better understand the importance of natural aerosol transmission routes of influenza virus and other respiratory viruses and to utilize infection control best practices to reduce nosocomial transmission of aerosolized viruses. Lindsley et al. (6) studied the generation of infectious influenza A virus from infected patients during coughs and exhalations by using the SKC BioSampler to assess the possible transmission modes. They found that both coughing and breathing are important activities in airborne influenza virus transmission and suggested that many of the viable influenza virus-containing particles might originate deep in the lungs. Lednicky and Loeb (7) isolated viable influenza A H3N2 virus from an SKC BioSampler and a personal cascade impactor sampler (PCIS) located up to 3.7 m from a sick subject. Milton et al. (8) found that fine particles (<5 μm, i.e., aerosol transmission mode) contained 8.8-fold more virus genomic equivalents than coarse particles (>5 μm, i.e., droplet transmission mode) and that surgical masks were not as effective for fine particles as for coarse particles. However, these studies were hindered by limitations in the air sampling and detection methods available for airborne viruses. Samplers commonly used for virus sampling, such as the cascade impactor or the BioSampler, are based on inertial impaction and thus are designed for collecting larger particles (>300 nm) (9), such as fungal spores and bacteria, and they are by design inefficient in collecting nanoparticles. For example, the commonly used SKC BioSampler has been shown to have less than 10% physical collection efficiency for aerosolized MS2 bacteriophage (28 nm), and a significant proportion of viruses become inactivated during the sampling process (10). The BioSampler has less than 8.6% collection efficiency for laboratory-generated aerosols of infectious influenza H1N1 virus (2009) (11). Although filters like Teflon filters and gelatin filters have high collection efficiency (99%) for a wide size range of particles from nanometers to micrometers, influenza viruses tend to become inactivated during collection on such filters (12). PCR methods, which have been widely used for virus quantification, provide a total count of the viral genomes (genome equivalents) but do not discriminate between genomes corresponding to viable versus nonviable viruses (13). These difficulties might account for the results of the study reported by Lindsley et al. (14), wherein influenza virus RNA was detected in 14 of the 30 test subjects yet infectious virus was isolated from only 2 subjects. Therefore, it is hard to know whether the low infectious virus recoveries reported in the literature are meaningful or due to poor collection methodologies for airborne viruses and/or inactivation of the viruses during the sampling process.

In this study, we evaluated the efficacy of the VIVAS for the collection of airborne influenza virus and other respiratory viruses in a student health care center during the course of a late-onset influenza virus outbreak. As our ongoing studies are focused on the collection of airborne influenza viruses, we subsequently carried out influenza virus genome sequence analyses to gain insights on which strains had been collected. The University of Florida (UF) has a highly vaccinated cohort of students and staff, and such virus genomic sequence information is useful for vaccine efficacy and infection control purposes and for virus surveillance efforts. The VIVAS uses a water-based condensation growth system to enable collection of aerosolized particles from 8 nm to 10 µm (15, 16). Importantly, enlargement of respired particles by water-based condensation occurs during natural breathing (12). We previously used the VIVAS for the successful collection of laboratory-generated influenza A virus aerosols, wherein the collection efficiency was more than 74% for viable influenza H1N1 virus (11). This high collection efficiency was attributed to the inherently gentler impaction with VIVAS of virus into the collection medium, which preserves infectivity, as well as the high physical collection efficiency for particles of a wide size range. With the VIVAS as a tool, we investigated whether infectious respiratory virus aerosols could be collected from the air of a student infirmary during an influenza outbreak as a model of its potential use in a real world environment.

## RESULTS

### Isolation and identification of viable viruses in aerosols collected 11 March 2016.

Viable human respiratory viruses were recovered by the VIVAS and the BioSampler in each of three separate air sampling intervals performed on 11 March, but no viruses were recovered in control runs performed with HEPA-filtered VIVAS and BioSampler air intakes. Cytopathic effects (CPE) consistent with those caused by influenza viruses were observed in MDCK cells beginning 6 days postinoculation (p.i.), suggesting the possibility that influenza A or B virus (or both) had been isolated (see Fig. S1A in the supplemental material). Moreover, syncytia consistent with those expected for human respiratory syncytial virus (RSV) were also observed in A549, LLC-MK2, and Vero cells regardless of trypsin content, but these effects were much more pronounced and easiest to detect in the cells in serum-free cell growth medium with added tosylsulfonyl phenylalanyl chloromethyl ketone (TPCK)-trypsin. The syncytia became evident beginning 8 days postinoculation of the LLC-MK2 cells with trypsin and 10 days later in trypsin-free LLC-MK2 cells. In contrast, no CPE were observed in mock-inoculated cells or in control runs with HEPA-filtered samplers. Though the onset varied according to sampling interval, in each case, the CPE were observed first in cells inoculated with collection media from the VIVAS.

10.1128/mSphere.00251-17.2 FIG S1  (A) MDCK cells in serum-free cell culture medium plus trypsin. (Left) Noninfected (mock-infected) MDCK cells 8 days postseed; the cell monolayer is intact and crowded. (Right) MDCK cells inoculated with collection medium from the VIVAS, sampling interval 1, experiment 1. Numerous rounded floating dead cells and large emptied areas of the growing surface are visible. Magnification, ×400. (B) Results of the solid-phase ELISAs. A negative-control reaction is shown in the left panel, whereas influenza A and B antigens were detected in cell culture medium (from MDCK cells inoculated with collection medium from VIVAS sampling interval 2, experiment 1). Download FIG S1, DOCX file, 1.1 MB. Copyright © 2017 Pan et al. 2017 Pan et al. This content is distributed under the terms of the Creative Commons Attribution 4.0 International license.

Solid-phase enzyme-linked immunosorbent assay (ELISA) results indicated that both influenza A and B viruses had been isolated from the VIVAS samples (Fig. S1B). Remarkably, reverse trancription-PCR (RT-PCR) indicated that influenza A virus subtypes H1 and H3 had been isolated, as well as Victoria-lineage influenza B virus. Similarly, RSV-A was identified in some of the cell cultures. The viruses detected per sampling interval are listed in Table 1; GenBank accession numbers for the influenza virus sequences are listed in Table S3, and those for RSV-A are summarized in Table S3.

10.1128/mSphere.00251-17.4 TABLE S1  Cell lines used for isolation of common culturable human respiratory viruses. Download TABLE S1, DOCX file, 0.02 MB. Copyright © 2017 Pan et al. 2017 Pan et al. This content is distributed under the terms of the Creative Commons Attribution 4.0 International license.

10.1128/mSphere.00251-17.5 TABLE S2  Primers for detection and subtyping of influenza A and B viruses. Download TABLE S2, DOCX file, 0.01 MB. Copyright © 2017 Pan et al. 2017 Pan et al. This content is distributed under the terms of the Creative Commons Attribution 4.0 International license.

10.1128/mSphere.00251-17.6 TABLE S3  GenBank accession numbers for influenza A and B virus sequences and RSV-A NS2 and N gene partial coding sequences (CDS), 11 March 2016. Download TABLE S3, DOCX file, 0.01 MB. Copyright © 2017 Pan et al. 2017 Pan et al. This content is distributed under the terms of the Creative Commons Attribution 4.0 International license.

**TABLE 1:** Viable viruses in aerosols collected on 11 March 2016

Sampling interval	HEPA filter	Air sampler	Virus isolated			
			Influenza A H1N1	Influenza A H3N2	Influenza B Victoria	RSV-A
1	No	BioSampler			+	
	No	VIVAS	+	+	+	+
2	Yes	BioSampler				
	Yes	VIVAS				
3	No	BioSampler				+
	No	VIVAS	+	+	+	+
4	Yes	BioSampler				
	Yes	VIVAS				
5	No	BioSampler	+			
	No	VIVAS	+	+	+	+
6	Yes	BioSampler				
	Yes	VIVAS				

Sequence data analyses revealed the H1N1 viruses belonged to HA subclade 6B.1, based on criteria mentioned in reference 17; the results are presented in Text S1 and Table S4 for the reference strains and other recent local H1N1 viruses we isolated from humans or from other air samplings in other studies. Similar analyses that were performed as outlined in reference 18 indicated that the influenza H3N2 viruses belonged to HA clade subclade 3C.2a. These viruses have amino acid changes at major immunogenic epitopes of the hemagglutinin (HA) protein (Table S5) and neuraminidase (NA) proteins (Table S5) relative to the vaccine strain. Finally, the influenza virus B strains were all Victoria lineage (clade 1A) viruses, as discussed in reference 19.

10.1128/mSphere.00251-17.1 TEXT S1 Additional information on the materials and methods for this study, including the cell culture media formulations for virus isolation, techniques for inoculation, maintenance, and observation of cell cultures, the GenMark respiratory virus panel, our methods for identification of RSV-A, and the influenza virus sequencing results from 11 March 2016. Download TEXT S1, DOCX file, 0.02 MB. Copyright © 2017 Pan et al. 2017 Pan et al. This content is distributed under the terms of the Creative Commons Attribution 4.0 International license.

10.1128/mSphere.00251-17.7 TABLE S4  Amino acid substitutions in the HA protein and deduced NA and M proteins of H1N1 virus from 11 March 2016. Download TABLE S4, DOCX file, 0.04 MB. Copyright © 2017 Pan et al. 2017 Pan et al. This content is distributed under the terms of the Creative Commons Attribution 4.0 International license.

10.1128/mSphere.00251-17.8 TABLE S5  Amino acid sequence differences in HA and NA of influenza H3N2 viruses in Gainesville, FL, 11 March 2016. Download TABLE S5, DOCX file, 0.04 MB. Copyright © 2017 Pan et al. 2017 Pan et al. This content is distributed under the terms of the Creative Commons Attribution 4.0 International license.

The RSV-A sequences were alike for the four isolates and were identical to those reported for contemporary RSV-A viruses in circulation in the United States (data not shown).

### Isolation and identification of viable viruses collected in aerosols on 28 March 2016.

**FIG 1:**
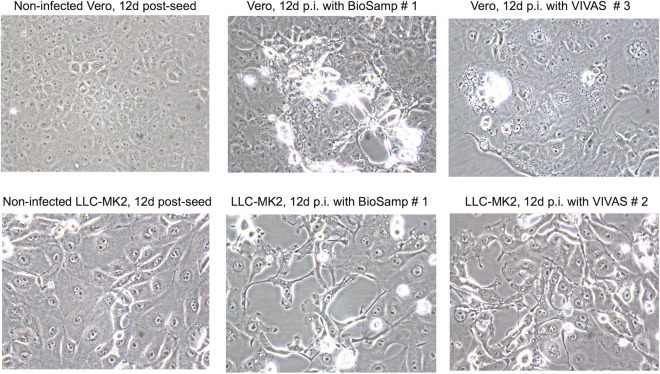
Mixed cytopathic effects in Vero E6 and LLC-MK2 cells inoculated with material collected 28 March. Original magnification, ×400.

A larger assortment of human respiratory viruses was isolated in air samplings performed on 28 March 2016 (Table 2). A GenMark readout for one of the mixed influenza A and B virus samples is shown in Fig. S2. Examples of cell cultures depicting virus-induced CPE are given in Fig. 1. Sequencing of the influenza viruses revealed that once again, H1N1 clade 6B.1, H3N2 clade 3C.2a, and Victoria-lineage influenza B viruses had been isolated, as during the 11 March 2016 air samplings. The RSV-A sequences were identical to those of the RSV-A sequences from 11 March. As this project was originally designed to answer whether influenza virus aerosols could be detected in the student health care center, the other viruses were not sequenced after they were identified based on a combination of diagnostic PCR or RT-PCR and GenMark analyses.

10.1128/mSphere.00251-17.3 FIG S2  Example of GenMark RT system analysis of MDCK cells inoculated with collection medium from VIVAS sampling interval 2, experiment 2. Positive detection of the genomic RNA of 2009 pandemic influenza H1, H3, and B viruses is shown. Other respiratory viruses were either not isolated or were inhibited by influenza viruses (or outcompeted by the influenza viruses) in these cells. Download FIG S2, JPG file, 1.1 MB. Copyright © 2017 Pan et al. 2017 Pan et al. This content is distributed under the terms of the Creative Commons Attribution 4.0 International license.

**TABLE 2:** Viable viruses in aerosols collected on 28 March, 2016

Sampling interval	HEPA filter	Air sampler	Virus isolated^a^									
			AdV	CoV 229E	CoV NL63	IFV A H1N1	IFV A H3N2	IFV B	HPIV-2	HPIV-3	HPIV-4a	RSV-A
1	No	BioSampler	+		+	+	+	+		+	+	+
	No	VIVAS	+		+	+	+	+	+	+	+	+
2	Yes	BioSampler										
	Yes	VIVAS										
3	No	BioSampler	+	+	+	+	+	+		+	+	+
	No	VIVAS	+	+	+	+	+	+		+	+	+
4	Yes	BioSampler										
	Yes	VIVAS										
5	No	BioSampler	+		+	+	+	+		+	+	+
	No	VIVAS	+	+	+	+	+	+		+	+	+
6	Yes	BioSampler										
	Yes	VIVAS										

a AdV, adenovirus; CoV, coronavirus; IFV, influenza virus; HPIV, human parainfluenza virus; RSV, respiratory syncytial virus.

### Isolation of only one type of virus from aerosols on 8 April 2016.

Unlike the results of the first two air sampling studies, neither influenza viruses nor RSV-A were isolated during the final air sampling studies at the student health care center. This result is in line with the observation that among the air sampling dates, the fewest number of patients were counted in the waiting room on 8 April 2016. Only 1 cell culture was positive for virus isolation, and that was for human metapneumovirus subtype A. Moreover, the virus was isolated from only one sample collected using the VIVAS that had been inoculated onto LLC-MK2 cells in serum-free medium with trypsin, and it required a >10-day incubation before CPE were formed in the infected cells (data not shown).

## DISCUSSION

We evaluated the virus aerosol collection capabilities of a novel air sampler (VIVAS) and a standard air sampler (BioSampler) in a student infirmary center during a late-onset influenza outbreak that spanned from mid-February to early April 2016. The experiments were performed three times a day on three separate days. In the first experiment (11 March 2016), infectious influenza A H1N1 and H3N2 viruses and influenza B viruses and RSV were present in the collection medium of the VIVAS in 3/3 air sampling intervals, but not in all of the BioSampler samples (Table 1). In the second experiment (28 March 2016), influenza A and B viruses and RSV were collected by both air samplers at each air sampling interval (Table 2), though it was noted that influenza virus-specific CPE were detected earlier in MDCK cells inoculated with VIVAS collection medium. No influenza virus or RSV was collected in the third experiment (8 April 2016). Other human respiratory viruses were also collected in the second experiment (Table 2), whereas human metapneumovirus was detected in one sample collected using the VIVAS in the third experiment.

In previous studies, the VIVAS and similar devices were compared to the BioSampler for the collection of laboratory-generated virus-containing particles from below 100 nm to larger than 10 μm (20, 21). In those study, the VIVAS outperformed the BioSampler for the collection of influenza virus and bacteriophage MS2 aerosols, due to gentle impaction and size amplification (20, 22). Results from this study also indicated that the VIVAS is better than the BioSampler for the collection of virus aerosols and preservation of virus viability in a real world environment. Possible reasons include the following: (i) the gentler impaction of the VIVAS is less damaging to viruses during the collection process, and (ii) the VIVAS has a higher physical collection efficiency than the BioSampler for particles as small as 8 nm due to the amplification results.

Apart from choice of a proper sampling location, the presence of an “emitting source” favors successful detection of the agent whose transmission is under evaluation. In our experiments, the location chosen was a student infirmary, the agent was influenza A or B virus, and the emitting source was a person with an active respiratory infection who was breathing, coughing, talking, and/or sneezing. Our findings were consistent with the previously mentioned items and information regarding the late-onset influenza season of 2016 provided by the State of Florida Department of Health (23), and they reinforce the notion that successful detection of virus aerosols is favored by location selection (i.e., virus aerosols should be highest at enclosed sites with numerous sick persons) and timing of tests (i.e., the likelihood of finding virus aerosols is higher during outbreaks of respiratory infection). In another three sets of air samplings performed using both the BioSampler and the VIVAS on three different days after the influenza outbreak (19 and 26 August and 9 September 2016) in a small classroom in UF’s Black Hall, no viable respiratory viruses were isolated (data not shown). As the sampling sites were at least 2 m from the patients, our results support the notion that an aerosol transmission mode is possible for influenza viruses.

Various studies have indicated that in temperate climates, influenza outbreaks typically occur in the late fall or early winter, when it is cold and the humidity is low (24, 25). The late-onset influenza outbreak of 2016 in Florida spanned from February to about the beginning of April (19). It is thus likely that influenza viruses were not present in the breathing air of the infirmary (or were present at extremely low concentrations) on 8 April, as the outbreak had been declared over, and few students sought medical treatment for respiratory infections that day. In contrast, the influenza season was near peak levels from 6 to 12 March (23), and influenza viruses were isolated in our air samplings on 11 and 28 March.

Viral genomic sequence analyses indicated that influenza H1N1 viruses were from the pandemic H1N1 year 2009 lineage [A(H1N1)pdm09]. However, unlike influenza virus A/California/07/2009(pdmH1N1), which is from pdm09 HA clade 1 and was used in the Northern Hemisphere influenza virus vaccines of 2015–2016 (12, 26, 27), the viruses isolated in this work were from pdm09 HA clade 6B.1 (Text S1). The signature amino acid substitutions that define subgroup 6B viruses are D97N, S185T, and A256T for HA1 and E47K and S124N for HA2. Additional variations that had been observed in 2016 included S162K, D168N, K170E, R205K, A215G, and E235D. As of February 2016, the pdmH1N1(09) viruses in all Europäische Union-European Economic Area countries had the additional substitutions K163Q, A256T, and K283E in HA1 and E172K in HA2, and newer strains also had P83S and I321V substitutions in HA1 as well (28). The situation in North America was similar. Whereas various virus clusters emerged within clade 6B, two dominant subclades, 6B.1 and 6B.2, were in wide circulation. Viruses in subclade 6B.1 have HA1 amino acid substitutions S84N and S162N (which result in the formation of a new potential glycosylation motif at residues 162 to 164 of HA1) and I216T. Subclade 6B.2 viruses have the HA1 amino acid substitutions V152T and V173I (28–30). As shown in Table S4, there were changes at key amino acid positions of the HA protein of the H1N1 viruses in this work relative to the vaccine strain, and the same changes were observed in H1N1 strains from the same time period that had been isolated from humans (data not shown) (31). Importantly, changes also occurred in the amino acid sequence of the NA protein (Table S4) and matrix (M) protein (Table S4). Similarly, seven genetic groups based on the HA gene have been defined for A(H3N2) viruses since 2009, and contemporary H3N2 viruses belong to clade 3C, which has three subdivisions, 3C.1, 3C.2, and 3C.3. The virus strain of the 2015–2016 Northern Hemisphere vaccine was A/Texas/50/2012(H3N2), which is an HA subclade 3C.1 virus. Subclade 3C.2a viruses have been dominant worldwide as of May 2016. The HA protein of 3C.2 viruses contains the amino acid substitutions N145S in HA1 and D160N in HA2, whereas subclade 3C.2a viruses contain the following amino acid substitutions at the major antigenic epitopes of HA1: N144S (resulting in loss of a potential glycosylation site), N145S, F159Y, and K160T (in the majority of viruses, these changes result in the gain of a potential glycosylation site), and N225D. Subclade 3C.2a viruses also contain (at other epitopes) L3I and Q311H in HA1 and D160N in HA2 (18, 28). The H3N2 viruses of our study were HA subclade 3C.2a (Table S5) and also had amino acid changes in their NA protein (Table S5). Finally, the influenza B viruses were Victoria lineage, whereas the commonly used influenza trivalent vaccine of 2015–2016 had a Yamagata-lineage strain. Given that UF has a highly vaccinated student and worker population, these findings raise the question of whether some/most of the influenza virus vaccines that had been used for the 2015–2016 season were not a good match for the influenza viruses in circulation in early 2016 in Florida.

There are some limitations to this pilot study. First, the VIVAS and the BioSampler were used to collect virus aerosols, but particle sizes were not determined. It is well-known that respiratory viruses can be present in different-sized airborne particles, and in particular, that influenza A virus can be detected in coughs and exhalations. Some of the influenza viruses are found in particle sizes termed “fine” particles, which stay airborne much longer than larger particles and can travel much longer distances, although the importance of these particles in virus transmission remains controversial (7, 13, 32, 33). In this study, the VIVAS and the BioSampler were located at least 2 m from seated patients, and viable (infectious) influenza A and B viruses were isolated. Other respiratory viruses were also isolated, suggesting that patients with other types of respiratory viruses also produce small aerosols that contain infectious viruses. Future studies should consider particle size characterization for better identification of the possible effects of exposure to virus and particle deposition in the respiratory track. Second, only two locations at the health care facility were tested for this study and the sample size was small. Further studies sampling more locations and more times would be useful in quantifying the ability of the VIVAS to sample different kinds of infectious viruses than does the BioSampler.

This study suggests that the VIVAS performed well for the collection of virus aerosols and the preservation of virus viability in a real world environment. An additional benefit was that virus aerosols were collected onto a small volume of collection medium, which simplified downstream operations, including storage, transport, and virus isolation. Moreover, many types of airborne viruses, including influenza A H1N1 and H3N2 and B viruses, adenovirus, coronavirus, human parainfluenza virus, and respiratory syncytial virus, were successfully collected by the VIVAS, indicating that the device is potentially useful for the general surveillance of airborne viruses.

## MATERIALS AND METHODS

All procedures were reviewed and approved by the director of the student health center. Approval by an institutional review board (IRB) was not necessary because human subjects were not studied and could not be identified and the sources of the viruses detected could not be tracked.

### Health care facility.

The Student Health Care Center at the University of Florida (Gainesville, FL) is a freestanding building that has its own heating, ventilation, and air conditioning (HVAC) system. During the study, indoor air temperatures were maintained at around 71°F (22°C) on the first floor and 73°F (23°C) on the second floor, whereas the relative humidity ranged from 44% to 46% on both floors. Air samplings at a small classroom in another UF building (Black Hall) were conducted for comparison. Black Hall also has its own HVAC system, wherein indoor temperatures were typically around 73°F (23°C) and relative humidity around 47% when this work was performed.

### Sampling dates.

Air samplings at the Student Health Care Center were performed on 11 March, 28 March, and 8 April 2016, as there was an influenza outbreak in Gainesville in March 2016. Air samplings in Black Hall were performed on 19 August, 26 August, and 9 September 2016.

### Aerosol collection system.

**FIG 2:**
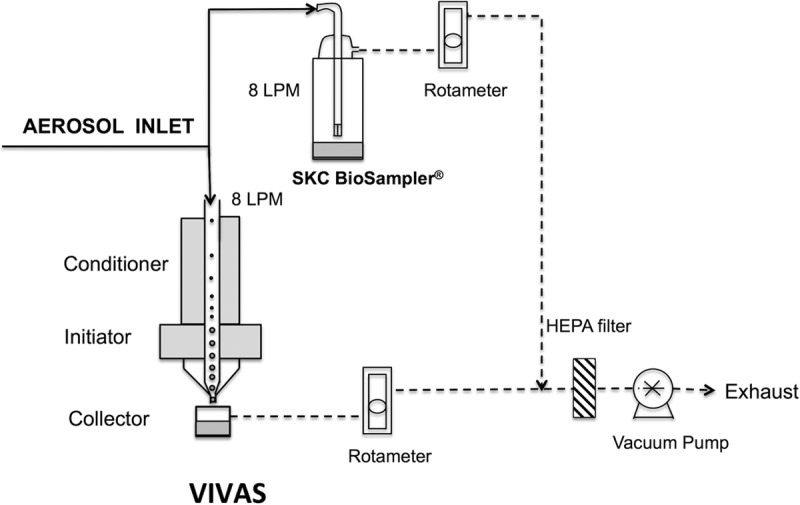
Schematic diagram of the air sampling system.

Ambient virus aerosol particles from both the Student Health Care Center and Black Hall were collected using the VIVAS and an SKC BioSampler. The collection system is schematically depicted in Fig. 2. The VIVAS collection mechanism was previously described (20); briefly, after passage through a cool temperature conditioner, the initiator activates and grows particles as small as 8 nm into droplets greater than 2 µm in diameter (Fig. 2). The enlarged particles are subsequently directed through a set of 32 nozzles of 0.66-mm diameter for gentle collection onto 1.5 ml of collection medium consisting of phosphate-buffered saline (PBS) plus 0.5% (wt/vol) bovine serum albumin (BSA) fraction V in a 35-mm-diameter petri dish. The BioSampler is presently considered the reference air sampler for the collection of airborne bacteria and fungi, and it has been used in attempts to collect virus aerosols (7, 34). During its use, intake air is diverted through three 0.63-mm tangential nozzles above the collection medium, resulting in a swirling airflow; this airflow reduces impaction forces on particles that are deposited onto the collection medium. The same collection medium was used with the BioSampler, but by necessity the volume in the BioSampler was 20 ml. To avoid discrepancies in virus concentrations due to sampling location, the inlets of the VIVAS and the BioSampler were bound together. Both the VIVAS and the BioSampler were operated at a flow rate of 8 liters/min, as this sampling rate was more effective for both air samplers for the collection of virus aerosols and maintenance of the virus infectivity compared with the standard flow rate of 12.5 liters/min (35, 36). To reduce noise, the sampling pump (Welch model 2014-B01) was placed within a covered box.

### Air sampler placement.

**FIG 3:**
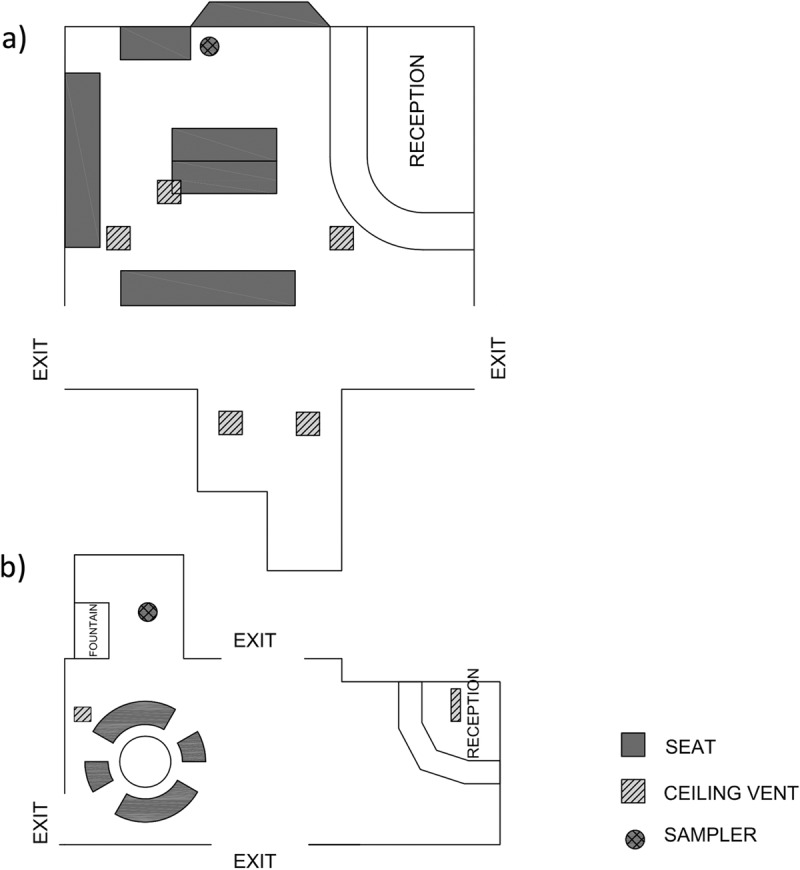
Schematic layout of the student infirmary lobby. (a) Second floor; (b) first floor.

The layout of the study areas, their ceiling vents, and the air sampler positions in the Student Health Care Center are shown in Fig. 3. The first sampling (11 March) was conducted in the first floor lobby, near the center’s reception desk (Fig. 3b). As the air was sampled, it was observed that few people sat near the receptionist’s desk but many took a drink at the fountain. During the second and third air samplings (28 March and 8 April), the air samplers were positioned within a waiting room on the second floor (Fig. 3a). On 28 May, the waiting room was visibly crowded, and as people waited for medical attention, many were observed to be coughing and sneezing, which are obvious signs of respiratory infections. In contrast, relatively few patients were present on 8 April. Air samplings in the classroom in Black Hall were conducted right after classes, with few if any students remaining in the room. The inlets of the air samplers were set at a height of 1.2 m oriented horizontal to the ground and facing the people, which approximates the average height of a seated adult person. Negative-control runs were also performed with a high-efficiency particulate air (HEPA) filter installed at the inlet of the samplers. A collection time of 60 min was used to sample about 480 liters of air. Air sampling and negative-control runs were conducted alternatively. Upon completion of air samplings, the pump was shut off, the VIVAS and the BioSampler were disconnected, and collection medium in each apparatus was aseptically transferred into sterile 50-ml conical polypropylene tubes, which were immediately placed in an insulated box with ice packs for transport to the laboratory. Similar sampling procedures were followed for the sampling at Black Hall, wherein the samplers were placed at the front corner of the classroom close to the podium.

### Virology laboratory.

Virology work was performed in a biosafety level 2 enhanced laboratory at the UF.

### Air sampler collection medium volume reduction and adjustment.

To standardize volumes and obviate large-scale cell cultures for virus isolation attempts, air sampler collection medium samples were concentrated using Amicon Ultra-15 centrifugal filter units with Ultracel-100 membranes with a molecular mass cutoff of 100 kDa (Millipore, Bedford, MA) at 4,000 × *g* for 20 min to a volume of approximately 400 μl, the volumes were adjusted to 500 µl by addition of collection medium, and the concentrate was stored at −80°C until virus isolation in cell cultures was attempted (7).

### Cell lines.

Emphasis was on the isolation of influenza viruses and genetic analyses thereof, but lesser efforts were nevertheless exerted to isolate other common “culturable” human respiratory viruses to gain insights on the general utility of the VIVAS for virus aerosols. The term “culturable” refers to those viruses that can be isolated and propagated using standard cell lines and methods. To favor isolation of a wide variety of respiratory viruses, concentrated air sample collection media were inoculated onto a variety of readily available (“standard”) cell lines obtained from the American Type Culture Collection (ATCC) for virus isolation attempts, including the following: A549 (CCL-185), HeLa (CCL-2), LLC-MK2 (CCL-7), MDCK (CCL-34), MRC-5 (CCL-171), NCI-H292, and Vero E6 (CRL-1586). Common human respiratory viruses that can be isolated using these cells are shown in Table S1 (T. Bonny and J. Lednicky, unpublished data). All the cell lines were propagated as monolayers at 37°C and 5% CO_2_ in advanced Dulbecco’s modified Eagle’s medium (aDMEM) or Eagle’s minimal essential medium (EMEM) (both from Invitrogen, Carlsbad, CA), as appropriate per cell line. Prior to the preparation of seed stocks, each cell line was treated for 3 weeks with plasmocin and verified free of mycoplasma DNA by PCR (37).

### Inoculation and identification of human respiratory viruses.

After thawing on ice, equal aliquots (~50 µl) of the archived concentrated air sampler collection media were inoculated directly without prefiltration onto newly confluent cells in 6-well plates. The inoculated cells were incubated at 35°C, and plates were monitored daily for signs of virus-induced CPE (i.e., formation of visible changes in the appearance of the nuclei of infected cells, together with the formation of focal enlarged granular cells or nonspecific cell deterioration, followed by detachment of the swollen cells from the growth surface), with refeeds performed every 3 days. Noninfected cells were maintained and refed in parallel for comparison. Cells were maintained and observed for a total of 30 days before being considered negative for virus isolation. Viruses were identified based on cell tropism (Table S1), type of CPE they induced, use of the GenMark respiratory virus panel, a solid-phase ELISA for influenza A and B viruses, group- and/or virus species-specific PCR or RT-PCR, and sequencing of virus-specific PCR amplicons or complete virus genomes.

### GenMark respiratory virus panel.

A GenMarkDx* *multiplex PCR eSensor XT-8 respiratory viral panel (eSensor RVP; GenMark Diagnostics, Inc., Carlsbad, CA) was used to screen spent cell growth media for the genomic DNA or RNA of respiratory viruses, according to the manufacturer’s instructions. This technique detects the genomic material of influenza A virus (including subtypes H1 and H3), influenza A virus 2009 H1N1, influenza B virus, respiratory syncytial viruses A and B, parainfluenza viruses 1, 2, 3, and 4, human metapneumovirus, adenoviruses B/E and C, coronaviruses 229E, -NL63, -HKU1, and -OC43, and human rhinoviruses A and B.

### Rapid detection of influenza A and B viruses in cell cultures.

A commercial solid-phase ELISA (QuickVue influenza A and B kit; Quidel Corp., San Diego, CA) was used per the manufacturer’s instructions to quickly detect influenza virus in the spent media of cell cultures that exhibited typical influenza virus-induced CPE (7). The ELISA did not distinguish between influenza A virus types H1 and H3.

### Identification of influenza virus types and subtypes and genomic sequencing.

After detection of virus using the QuickVue influenza A and B kit and the GenMark system, viral RNA was purified from the virus particles in spent MDCK cell growth medium, and preliminary analyses were performed by RT-PCR using the primers listed in Table S2 to establish virus type and subtype. Sequencing of influenza A virus genomic segments 4 (HA gene), 6 (NA gene), and 7 (M2 and M1 genes) and influenza B virus segments 4 (HA gene), 6 (NB glycoprotein and NA genes), and 7 (M1 and BM2 genes) was accomplished following previously described methods (18, 38).

### Identification of RSV-A.

Both RSV subtypes A and B induce the formation of syncytia in LLC-MK2 and Vero E6 cells and, to a lesser extent, in A549 cells. In general, the RSV-induced CPE is first detected in LLC-MK2 cells, then in Vero E6 cells, and lastly in A549 cells, regardless of the presence or absence of trypsin. However, syncytia are generally observed earlier in RSV-infected cell cultures in which trypsin is present. Since many viruses induce the formation of syncytia, confirmation through additional tests are required. The syncytium-forming viruses were identified as RSV-A by analyses of vRNA purified 12 days postinfection from virus particles in the spent cell growth medium of TPCK-containing LLC-MK2 cells (Text S1) by using the GenMark system and by RT-PCR followed by sequencing of the PCR amplicons.

### Identification of miscellaneous respiratory viruses.

Adenovirus C (type 5), human parainfluenza viruses 2, 3, and 4a, human coronaviruses 229E and NL63, and human metapneumovirus were identified using the GenMark system and by using published PCR-based methods (further details are available upon request).
