# Supplementary material for: Collection of Viable Aerosolized Influenza Virus and Other Respiratory Viruses in a Student Health Care Center through Water-Based Condensation Growth
Source: mSphere. 2017 Oct 11;2(5):e00251-17. doi: 10.1128/mSphere.00251-17 (PMC5636224; doi:10.1128/mSphere.00251-17)
Supplement: FIG S1 [file sph005172380sf1.docx]

Fig. S1

(A)


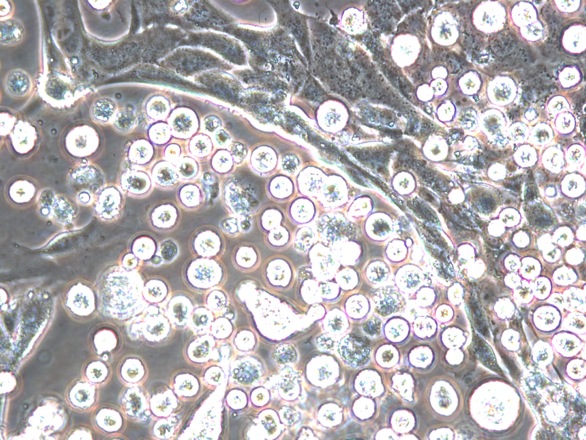

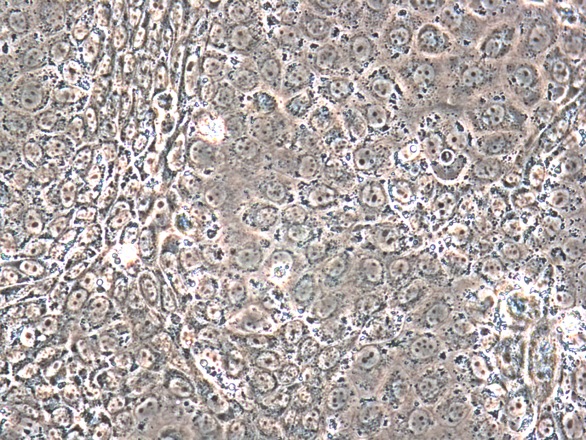


Mock-infected MDCK cells, 8 days post-seed

MDCK cells + VIVAS-1 inoculum, 8 days p.i.

(B)


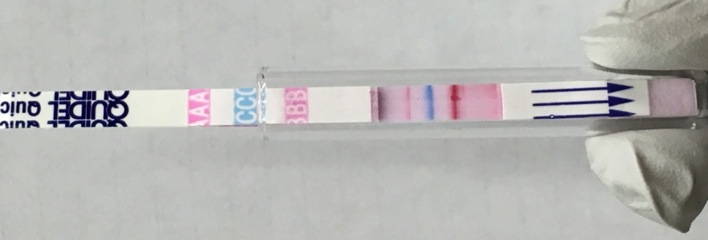

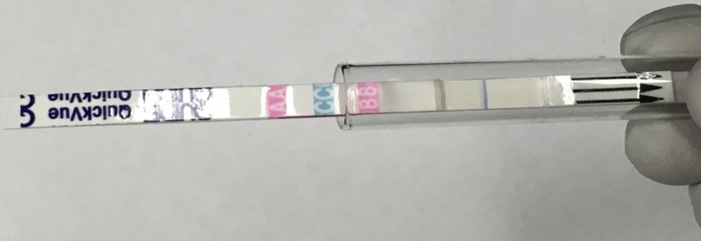


Reference line

Negative Control

Influenza A + B virus

Reference line

Influenza A

Influenza B
