# Supplementary material for: Collection of Viable Aerosolized Influenza Virus and Other Respiratory Viruses in a Student Health Care Center through Water-Based Condensation Growth
Source: mSphere. 2017 Oct 11;2(5):e00251-17. doi: 10.1128/mSphere.00251-17 (PMC5636224; doi:10.1128/mSphere.00251-17)
Supplement: TABLE S1 [file sph005172380st3.docx]

Table S1. Cell lines for the isolation of common culturable human respiratory viruses.

| Human respiratory virus | | Cell line | | | | | | |
| --- | --- | --- | --- | --- | --- | --- | --- | --- |
|  |  | **A549** | **HeLa** | **LLC-MK2** | **MDCK** | **MRC-5** | **NCI-H292** | **Vero E6** |
| Adenoviruses | | ++^a^ | + |  | some | + |  | + |
| Coronavirus 229E | |  |  |  |  | ++ |  |  |
| Coronavirus NL63 | |  |  | ++ | +/- |  |  | + |
| Coronavirus OC43 | |  |  |  |  | +/- |  |  |
| Influenza A and B viruses | | some |  | + | ++ |  | some | + |
| Metapneumovirus | |  |  | ++ |  |  |  | + |
| Parainfluenzavirus 1 | |  |  | ++ |  | +/- |  | + |
| Parainfluenzavirus 2 | | + |  | ++ |  | +/- |  | + |
| Parainfluenzavirus 3 | | + |  | ++ |  | + | + | + |
| Parainfluenzavirus 4a | | +/- |  | ++ |  | +/- |  | + |
| Parainfluenzavirus 4b | |  |  | ++ |  |  |  | + |
| Respiratory syncytial virus A | | + | + | ++ |  | +/- | ++ | ++ |
| Respiratory syncytial virus B | |  |  | ++ |  | +/- | ++ | ++ |
| Picornavirus group | Coxsackievirus A |  |  | + |  | + | ++ |  |
|  | Coxsackievirus B | ++ | ++ | ++ | +/- | + | ++ | ++ |
|  | Echovirus | +/- | + | ++ |  | ++ |  | + |
|  | Enterovirus (most) | ++ |  | ++ |  | ++ | ++ | + |
|  | Enterovirus 71 | + |  | ++ |  | ++ |  | + |
|  | Enterovirus D68 | ++ |  | + |  | + |  | +/- |
|  | Parechovirus |  | ++ |  |  |  |  | + |
|  | Rhinovirus A, B | + | + | + |  | ++ |  | + |

^a^++; Preferred indicator cell line.
