# Supplementary material for: Collection of Viable Aerosolized Influenza Virus and Other Respiratory Viruses in a Student Health Care Center through Water-Based Condensation Growth
Source: mSphere. 2017 Oct 11;2(5):e00251-17. doi: 10.1128/mSphere.00251-17 (PMC5636224; doi:10.1128/mSphere.00251-17)
Supplement: TABLE S2 [file sph005172380st4.docx]

Table S2. Primers for the detection of and subtyping of influenza A and B viruses.

| **Influenza virus type/subtype** | **Gene fragment** | **Primer** | **Sequence (5’ – 3’)** | **Reference** | **Amplicon size (bp)** |
| --- | --- | --- | --- | --- | --- |
| Influenza A | All genes (F) | Uni12W | AGCRAAAGCAGG | WHO^a^ | Not applicable |
|  | All genes (R) | UniR | AGTAGAAACAAGG | Hoffman *et al*.^b^ |  |
| A(H1N1)2009 | HA-5’ (H1) | HKU-SWF | GAGCTCAGTGTCATCATTTGAA | WHO^a^ | 173 |
|  |  | HKU-SWR | TGCTGAGCTTTGGGTATGAA |  |  |
|  |  | UFH1-JLR | GGTTGAGCTTTGGGTATGAA | J. Lednicky^c^ |  |
|  | NA-3’(N1) | N1F401 | GGAATGCAGAACCTTCTTCTTGAC | WHO^a^ | 1073 |
|  |  | NARUc | ATATGGTCTCGTATTAGTAGAAACAAGGAGTTTTTT |  |  |
| A(H3N2) | HA-3’(H3) | H3A1F3 | TGCATCACTCCAAATGGAAGCATT | WHO^a^ | 863 |
|  |  | HARUc | ATATCGTCTCGTATTAGTAGAAACAAGGGTGTTTT |  |  |
|  | NA-3’(N2) | N2F387 | CATGCGATCCTGACAAGTGTTATC | WHO^a^ | 1082 |
|  |  | NARUc | ATATGGTCTCGTATTAGTAGAAACAAGGAGTTTTTT |  |  |
| B Victoria-lineage | HA | Bvf224 | ACATACCCTCGGCAAGAGTTTC | WHO^a^ | 284 |
|  |  | Bvr507 | TGCTGTTTTGTTGTTGTCGTTTT |  |  |
| B Yamagata**-** lineage | HA | Byf226 | ACACCTTCTGCGAAAGCTTCA | WHO^a^ | 388 |
|  |  | Byr613 | CATAGAGGTTCTTCATTTGGGTTT |  |  |

^a^WHO. WHO information for molecular diagnosis of influenza virus.

(http://www.who.int/influenza/gisrs_laboratory/molecular_diagnosis_influenza_virus_humans_update_201403rev201505.pdf?ua=1).

^b^Hoffmann E, Stech J, Guan Y, Webster RG, Perez DR. 2001. Universal primer set for the full-length amplification of all influenza A viruses. Arch Virol 146 (12), 2275-2289.

^c^J. Lednicky, unpublished.
