# Supplementary material for: Collection of Viable Aerosolized Influenza Virus and Other Respiratory Viruses in a Student Health Care Center through Water-Based Condensation Growth
Source: mSphere. 2017 Oct 11;2(5):e00251-17. doi: 10.1128/mSphere.00251-17 (PMC5636224; doi:10.1128/mSphere.00251-17)
Supplement: TABLE S3 [file sph005172380st5.docx]

Table S3. GenBank accession numbers for Influenza A and B virus sequences, and *Respiratory syncytial virus*-A NS2 and N gene partial cds sequences, of viruses collected March 11, 2016.

| **Influenza virus** | **GenBank Accession #** | | |
| --- | --- | --- | --- |
|  | ***Hemagglutinin* gene** | ***NB* and/or *Neuraminidase* genes** | ***Matrix* genes** |
| A/envr/GNVL/01/2016 (H1N1) | KX398060.1 | KX398064.1 | KX398068.1 |
| A/envr/GNVL/02/2016 (H1N1) | KX398061.1 | KX398065.1 | KX398069.1 |
| A/envr/GNVL/03/2016 (H1N1) | KX398062.1 | KX398066.1 | KX398070.1 |
| A/envr/GNVL/04/2016 (H1N1) | KX398063.1 | KX398067.1 | KX398071.1 |
|  |  |  |  |
| A/envr/GNVL/01/2016 (H3N2) | KX398081.1 | KX398084.1 | KX398087.1 |
| A/envr/GNVL/02/2016 (H3N2) | KX398082.1 | KX398085.1 | KX398088.1 |
| A/envr/GNVL/03/2016 (H3N2) | KX398083.1 | KX398086.1 | KX398089.1 |
|  |  |  |  |
| B/envr/GNVL/01/2016 | KX398072.1 | KX398075.1 | KX398078.1 |
| B/envr/GNVL/02/2016 | KX398073.1 | KX398076.1 | KX398079.1 |
| B/envr/GNVL/03/2016 | KX398074.1 | KX398077.1 | KX398080.1 |
|  |  |  |  |
| ***Respiratory syncytial virus*-A** | **NS2 and N gene partial cds** | | |
| RSVA/Environmental Air/Gainesville/UF-1/2016 | KX431988.1 | | |
| RSVA/Environmental Air/Gainesville/UF-2/2016 | KX431989.1 | | |
| RSVA/Environmental Air/Gainesville/UF-3/2016 | KX431990.1 | | |
| RSVA/Environmental Air/Gainesville/UF-4/2016 | KX431991.1 | | |
