# Supplementary material for: Collection of Viable Aerosolized Influenza Virus and Other Respiratory Viruses in a Student Health Care Center through Water-Based Condensation Growth
Source: mSphere. 2017 Oct 11;2(5):e00251-17. doi: 10.1128/mSphere.00251-17 (PMC5636224; doi:10.1128/mSphere.00251-17)
Supplement: TEXT S1 [file sph005172380s8.docx]

**Supplemental MATERIALS AND METHODS**

**1. Cell culture media formulations for virus isolation**. Some respiratory viruses such as Influenza A viruses and various paramyxoviruses are easiest to propagate in the presence of trypsin or similar protease in the cell culture medium. However, FBS has a trypsin inhibitor, and cell lines vary in their susceptibility to trypsin levels. Therefore, for the isolation and propagation of viruses that require trypsin, FBS is typically omitted when possible and the concentration of trypsin adjusted as needed per cell line. For this work, ten combinations of cells and culture condition were used: Six cell lines in complete cell growth medium plus serum (A549, HeLa, LLC-MK2, MDCK, MRC-5, NCI-H292, and Vero E6), and four cell lines in serum-free media plus L-1-tosylamido-2-phenylethyl chloromethyl ketone (TPCK)-treated mycoplasma- and extraneous virus-free trypsin (Worthington Biochemical Company, Lakewood, NJ). TPCK-treated trypsin concentrations were: 0.12 µg/ml for A549, 0.25 µg/ml for LLC-MK2 and Vero cells, and 2.0 µg/ml for MDCK cells.

**2. Inoculation, maintenance, and observation of cell cultures**. After thawing on ice, equal aliquots (~ 50 µl) of the archived concentrated air sampler collection media were inoculated directly without pre-filtration onto newly confluent cells in 6-well plates. Importantly, pre-filtration through a 0.45 µm pore-size, as practiced by many to remove bacterial and fungal contaminants and particulates, was not performed. This is because some human respiratory viruses are pleomorphic and/or filamentous (such as wild-type human influenza A viruses), with lengths that exceed 0.45 µm, and these can be trapped by the filters. The inoculated cells were incubated at 35°C, and observed daily for signs of virus-induced cytopathic effects (CPE), with re-feeds performed every 3 days. Non-infected cells were maintained and re-fed in parallel for comparison. Cells were maintained and observed for a total of 30 days before being considered negative for virus isolation. In the event the virus that any of the cultures were contaminated by yeast or filamentous fungi, amphotericin B (Fungizone, Invitrogen) was available as a cell culture medium supplement, as needed.

**3. GenMark Respiratory Virus Panel.** The GenMark Dx multiplex PCR eSensor XT-8 Respiratory Viral Panel (eSensor RVP; GenMark Diagnostics, Inc., Carlsbad, CA, USA) was used to screen spent cell growth media for the genomic DNA or RNA of respiratory viruses according to the manufacturer’s instructions. The instrument detects the genomic material of influenza A virus subtypes H1 and H3, and separately, signals detection of pandemic 2009 influenza A virus H1N1, and influenza B virus, respiratory syncytial viruses A and B, parainfluenza viruses 1, 2, 3, and 4, human metapneumovirus, adenoviruses B/E and C, coronaviruses (229E, -NL63, -HKU1, -OC43), and human rhinoviruses A and B. Briefly, in the case of viral genomic RNA (vRNA), the extracted nucleic acid is reverse transcribed and amplified using viral specific primers with an RT-PCR enzyme mix. The amplified DNA is converted to single-stranded DNA via exonuclease digestion and is combined with a signal buffer containing ferrocene-labeled signal probes that are specific for the different viral targets. A signal in nanoAmperes (nA) is provided; signals higher than a threshold value are considered positive.

**4. Detection and partial genomic sequencing of *Respiratory syncytial virus subtype A* (RSV-A).** Both RSV subtypes A and B induce the formation of syncytia in LLC-MK2 and VeroE6 cells, and to a lesser extent, in A549 cells. In general, the RSV-induced CPE are first detected in LLC-MK2 cells, then in Vero E6, and lastly in A549 cells, regardless of the presence or absence of trypsin. However, syncytia are generally observed earlier in RSV-infected cell cultures in the presence of trypsin. Since many viruses induce the formation of syncytia, confirmation through additional tests are required. The syncytium-forming viruses were identified as RSV-A by analyses of vRNA purified 12 days post-infection from virus particles in the spent-cell growth media of TPCK-containing LLC-MK2 cells using the GenMark system, and by RT-PCR followed by sequencing of the PCR amplicons. Weak 80-bp amplicons specific for RSV-A resulted when RT-PCR was performed using forward primer RSA-U1137; 5’-AGATCAACTTCTGTCATCCAGCAA-3’ and reverse primer RSB-L1192 5’-GCACATCATAATTAGGAGTATCAAT-3’ (Briese *et al*.^1^), which target the RSV-A *nucleoprotein* (N) gene but were optimized for older RSV-A strains, suggesting these primers were not necessarily ideal for contemporary RSV-A strains in the USA. To compensate for nucleotide changes in contemporary RSV-A strains in the USA, the primers were slightly modified as: RSA-U1137-mod; 5’- AGATCAACTTCTATCATCCAGCAA-3’ and RSB-L1192-mod: 5’-agcacatcataattaggagtgtcaat-3’, and this improved RT-PCR detection, resulting in the formation of robust 81-bp amplicons. The entire 81-bp amplicon sequence was obtained by first re-amplifying a longer version (129 bp) with flanking primers RSV-81 Forward: 5’-caagttgaatgatacactcaacaa-3’, and RSV-81 Reverse: 5’- agaaacacattaataagttatgtg-3’, followed by sequencing to obtain non-ambiguous reads of the internal 81-bp target sequence. In contrast, primers for the detection of RSV-B (Briese *et al*.^1^) did not amplify a specific amplicon. Since short viral genomic sequences are not very informative, a longer (660-bp) RSV-A genomic sequence surrounding the 81-bp amplicon was therefore amplified and sequenced using primers RSV-A NS2-N For: 5’-. CATGATGGGTTCTTAGAATGC-3’ and RSV-A NS2-N Rev: 5’- CTGGAGCCACCTCTCCCATTTC-3’, and the 617-bp internal sequence thereof determined.

**Reference**

1. Briese T, Palacios G, Kokoris M, Jabado O, Liu ZQ, Renwick N, Kapoor V, Casas I, Pozo F, Limberger R, Perez-Brena P, Ju JY, Lipkin WI. 2005. Diagnostic system for rapid and sensitive differential detection of pathogens. *Emerg Infect Dis* *11* (2): 310-313.
