# Supplementary material for: Collection of Viable Aerosolized Influenza Virus and Other Respiratory Viruses in a Student Health Care Center through Water-Based Condensation Growth
Source: mSphere. 2017 Oct 11;2(5):e00251-17. doi: 10.1128/mSphere.00251-17 (PMC5636224; doi:10.1128/mSphere.00251-17)
Supplement: TABLE S4 [file sph005172380st6.docx]

Table S4A. Amino acid substitutions in the HA protein of H1N1 viruses from March 11, 2016.

| H1N1 Clade | Strain | Isolation Source | Key amino acid positions in HA1 | | | | | | | | | | | | Key amino acid positions in HA2 | | |
| --- | --- | --- | --- | --- | --- | --- | --- | --- | --- | --- | --- | --- | --- | --- | --- | --- | --- |
|  |  |  | 83 | 84 | 97 | 162 | 163 | 185 | 203 | 216 | 225 | 226 | 256 | 283 | 47 | 124 | 172 |
| 1 | California/07/2009 | Human | P | S | D | S | K | S | S | I | G | R | A | K | E | S | E |
| 6 | St. Petersburg/27/2011 | Human | S | S | D | S | K | S | T | I | G | R | A | K | K | S | E |
| 6A (subclade) | HongKong/5659/2012 | Human | S | S | N | S | K | T | T | I | G | R | A | K | K | N | E |
| 6C (subclade) | Massachusetts/10/  2013 | Human | S | S | N | S | N | S | T | I | G | R | A | E | K | N | K |
| 6B (subclade) | South Africa/3626/2013 | Human | S | S | S | S | Q | T | T | I | G | R | T | E | K | N | K |
| 6B | GNVL/08/2013 | Human | S | S | N | S | Q | T | T | I | G | R | T | E | K | N | K |
| 6B | GNVL/07/2014 | Human | S | S | N | S | Q | T | T | I | G | R | T | E | K | N | K |
| 6B | ENVR/GNVL/08/2015 | Environment | S | S | N | S | Q | T | T | I | G | R | T | E | K | N | K |
| 6B | ENVR/GNVL/12/2015 | Environment | S | S | N | S | Q | T | T | I | G | R | T | E | K | N | K |
| 6B | GNVL/01/2015 | Human | S | S | N | S | Q | T | T | I | G | R | T | E | K | N | K |
| 6B.1 | GNVL/01/2016 | Human | S | N | N | N | Q | T | T | T | G | R | T | E | K | N | K |
| 6B.1 | GNVL/02/2016 | Human | S | N | N | N | Q | T | T | T | G | R | T | E | K | N | K |
| 6B.1 | GNVL/03/2016 | Human | S | N | N | N | Q | T | T | T | G | R | T | E | K | N | K |
| 6B.1 | GNVL/04/2016 | Human | S | N | N | N | Q | T | T | T | G | R | T | E | K | N | K |
| 6B.1 | ENVR/GNVL/1/2016 | Environment | S | N | N | N | Q | T | T | T | G | R | T | E | K | N | K |
| 6B.1 | ENVR/GNVL/2/2016 | Environment | S | N | N | N | Q | T | T | T | G | R | T | E | K | N | K |
| 6B.1 | ENVR/GNVL/3/2016 | Environment | S | N | N | N | Q | T | T | T | G | R | T | E | K | N | K |
| 6B.1 | ENVR/GNVL/4/2016 | Environment | S | N | N | N | Q | T | T | T | G | R | T | E | K | N | K |

Table S4B. Amino acid changes in the deduced NA protein of H1N1 isolated March 11.

| Strain | amino acid positions in NA protein | | | | | | | | | | | | | | | | | | | | | | | |
| --- | --- | --- | --- | --- | --- | --- | --- | --- | --- | --- | --- | --- | --- | --- | --- | --- | --- | --- | --- | --- | --- | --- | --- | --- |
|  | 13 | 19 | 20 | 34 | 40 | 44 | 82 | 86 | 106 | 117 | 126 | 200 | 241 | 248 | 264 | 265 | 270 | 314 | 321 | 369 | 386 | 397 | 432 | 451 |
| California/07/  2009 | V | M | A | I | L | N | S | A | V | I | P | N | V | N | V | K | N | I | I | N | N | N | K | D |
| St. Petersburg/  27/2011 | V | M | A | I | L | N | S | A | I | I | P | N | I | D | V | K | N | I | I | K | N | N | K | D |
| HongKong/  5659/2012 | V | A | A | I | L | N | S | A | I | I | P | N | I | D | V | K | N | I | I | K | S | N | K | G |
| Massachusetts/  10/2013 | V | M | A | I | L | S | S | A | V | I | L | S | I | D | V | K | N | I | I | K | N | N | K | D |
| South Africa/  3626/2013 | V | M | A | V | L | N | S | A | V | I | P | S | I | D | V | K | N | I | V | K | N | N | K | D |
| GNVL/08/2013 | V | M | T | I | L | S | P | A | V | I | P | S | I | D | V | K | N | I | V | K | N | K | K | D |
| GNVL/07/2014 | V | M | A | V | L | S | S | A | V | I | P | S | I | D | V | K | N | I | V | K | K | N | K | D |
| ENVR/GNVL/08/2015 | V | M | A | V | I | S | S | V | V | M | P | S | I | D | V | K | N | I | V | K | K | N | E | D |
| ENVR/GNVL/12/2015 | V | M | A | V | I | S | S | V | V | M | P | S | I | D | V | K | N | I | V | K | K | N | E | D |
| GNVL/01/2015 | V | M | A | V | I | S | S | V | V | M | P | S | I | D | V | K | N | I | V | K | K | N | E | D |
| GNVL/01/2016 | I | M | A | V | I | S | S | A | V | I | P | S | I | D | I | R | K | M | V | K | K | N | E | D |
| GNVL/02/2016 | I | M | A | V | I | S | S | A | V | I | P | S | I | D | I | R | K | M | V | K | K | N | E | D |
| GNVL/03/2016 | I | M | A | V | I | S | S | A | V | I | P | S | I | D | I | R | K | M | V | K | K | N | E | D |
| GNVL/04/2016 | I | M | A | V | I | S | S | A | V | I | P | S | I | D | I | R | K | M | V | K | K | N | E | D |
| ENVR/GNVL/1/2016 | I | M | A | V | I | S | S | A | V | I | P | S | I | D | I | R | K | M | V | K | K | N | E | D |
| ENVR/GNVL/2/2016 | I | M | A | V | I | S | S | A | V | I | P | S | I | D | I | R | K | M | V | K | K | N | E | D |
| ENVR/GNVL/3/2016 | I | M | A | V | I | S | S | A | V | I | P | S | I | D | I | R | K | M | V | K | K | N | E | D |
| ENVR/GNVL/4/2016 | I | M | A | V | I | S | S | A | V | I | P | S | I | D | I | R | K | M | V | K | K | N | E | D |

Table S4C. Amino acid changes in the deduced M proteins of H1N1 from March 11, 2016.

| H1N1 Clade | Strain | Isolation Source | M1 protein M2 protein | | | | | | | |  |
| --- | --- | --- | --- | --- | --- | --- | --- | --- | --- | --- | --- |
|  |  |  | **80** | **85** | **167** | **192** | **208** | **230** |  | **21** | |
| 1 | California/07/2009 | Human | V | N | T | M | Q | K |  | D | |
| 6* | St. Petersburg/27/2011 | Human |  |  |  |  |  |  |  |  | |
| 6A (subclade) | HongKong/5659/2012 | Human | I | N | T | M | Q | K |  | D | |
| 6C (subclade) | Massachusetts/10/2013 | Human | I | S | T | V | Q | R |  | G | |
| 6B (subclade) | South Africa/3626/2013 | Human | I | N | T | V | Q | R |  | G | |
| 6B | GNVL/08/2013 | Human | I | N | T | V | Q | R |  | G | |
| 6B | GNVL/07/2014 | Human | I | N | T | V | Q | R |  | V | |
| 6B | ENVR/GNVL/08/2015 | Environment | I | N | A | V | Q | R |  | V | |
| 6B | ENVR/GNVL/12/2015 | Environment | I | N | A | V | Q | R |  | V | |
| 6B | GNVL/01/2015 | Human | I | N | A | V | Q | R |  | V | |
| 6B.1 | GNVL/01/2016 | Human | I | N | T | V | K | R |  | G | |
| 6B.1 | GNVL/02/2016 | Human | I | N | T | V | K | R |  | G | |
| 6B.1 | GNVL/03/2016 | Human | I | N | T | V | K | R |  | G | |
| 6B.1 | GNVL/04/2016 | Human | I | N | T | V | K | R |  | G | |
| 6B.1 | ENVR/GNVL/1/2016 | Environment | I | N | T | V | K | R |  | G | |
| 6B.1 | ENVR/GNVL/2/2016 | Environment | I | N | T | V | K | R |  | G | |
| 6B.1 | ENVR/GNVL/3/2016 | Environment | I | N | T | V | K | R |  | G | |
| 6B.1 | ENVR/GNVL/4/2016 | Environment | I | N | T | V | K | R |  | G | |

*Matrix protein sequence information not listed in publically accessible database.
