# Supplementary material for: Collection of Viable Aerosolized Influenza Virus and Other Respiratory Viruses in a Student Health Care Center through Water-Based Condensation Growth
Source: mSphere. 2017 Oct 11;2(5):e00251-17. doi: 10.1128/mSphere.00251-17 (PMC5636224; doi:10.1128/mSphere.00251-17)
Supplement: TABLE S5 [file sph005172380st7.docx]

**Table S5.** Amino acid sequence differences of deduced HA and NA proteins of influenza H3N2 viruses in Gainesville, Florida, March11, 2016.

| H3N2 Virus Strain | Amino Acid Sequence of Epitope A (aa 121 – 146) | | | | | | | | | | | | | | | | | | | | | | | | | | | | | | | | | | | | | Amino Acid Sequence of Epitope B1 (aa 155 – 163) | | | | | | | | | | | |
| --- | --- | --- | --- | --- | --- | --- | --- | --- | --- | --- | --- | --- | --- | --- | --- | --- | --- | --- | --- | --- | --- | --- | --- | --- | --- | --- | --- | --- | --- | --- | --- | --- | --- | --- | --- | --- | --- | --- | --- | --- | --- | --- | --- | --- | --- | --- | --- | --- | --- |
| H3N2 Consensus sequence^a^ | N | N | E | S | F | | N | W | | T | G | | V | T | | Q | N | | G | T | | S | S | A | | C | K | | R | R | | S | N | | N | S | | T | H | | L | K | F | | K | Y | | P | A |
| A/Texas/50/2012^b^ |  |  |  |  |  | |  |  | | N |  | |  |  | |  |  | |  |  | |  |  |  | |  | I | |  |  | |  |  | |  |  | |  |  | |  | N |  | |  |  | |  |  |
| A/GNVL/01/2014^C^ |  |  |  |  |  | |  |  | | A |  | |  |  | |  |  | |  |  | |  |  |  | |  | I | |  | G | |  |  | | S |  | |  |  | |  | N |  | |  |  | |  |  |
| A/GNVL/05/2014^d^ |  |  |  |  |  | |  |  | |  |  | |  |  | |  |  | |  |  | |  |  |  | |  | I | |  |  | |  | S | | S |  | |  |  | |  | N | Y | | T |  | |  |  |
| A/GNVL/06/2014^d^ |  |  |  |  |  | |  |  | |  |  | |  |  | |  |  | |  |  | |  |  |  | |  | I | |  |  | |  | S | | S |  | |  |  | |  | N | Y | | T |  | |  |  |
| A/GNVL/07/2014^d^ |  |  |  |  |  | |  |  | |  |  | |  |  | |  |  | |  |  | |  |  |  | |  | I | |  |  | |  | S | | S |  | |  |  | |  | N | Y | | T |  | |  |  |
| A/GNVL/08/2014^d^ |  |  |  |  |  | |  |  | |  |  | |  |  | |  |  | |  |  | |  |  |  | |  | I | |  |  | |  | S | | S |  | |  |  | |  | N | Y | | T |  | |  |  |
| A/GNVL/09/2014^e^ |  |  |  |  |  | |  |  | |  |  | |  |  | |  |  | |  |  | |  |  |  | |  | I | |  |  | |  | S | | S |  | |  |  | |  | N | Y | | T |  | |  |  |
| A/CH/9715293/2013^f^ |  |  |  |  |  | |  |  | | A |  | |  |  | |  |  | |  |  | |  |  | S | |  | R | |  | G | |  |  | | S |  | |  |  | |  | N | S | |  |  | |  |  |
| A/GNVL/01/2016^g^ |  |  |  |  |  | |  |  | |  |  | |  |  | |  |  | |  |  | |  |  |  | |  | I | |  |  | |  | S | | S |  | |  |  | |  | N | Y | | T |  | |  |  |
| A/GNVL/02/2016^g^ |  |  |  |  |  | |  |  | |  |  | |  |  | |  |  | |  |  | |  |  |  | |  | I | |  |  | |  | S | | S |  | |  |  | |  | N | Y | | T |  | |  |  |
| A/ENVR/GNVL/01/2016 |  |  |  |  |  | |  |  | |  |  | |  |  | |  |  | |  |  | |  |  |  | |  | I | |  |  | |  | S | | S |  | |  |  | |  | N | Y | | T |  | |  |  |
| A/ENVR/GNVL/02/2016 |  |  |  |  |  | |  |  | |  |  | |  |  | |  |  | |  |  | |  |  |  | |  | I | |  |  | |  | S | | S |  | |  |  | |  | N | Y | | T |  | |  |  |
| A/ENVR/GNVL/03/2016 |  |  |  |  |  | |  |  | |  |  | |  |  | |  |  | |  |  | |  |  |  | |  | I | |  |  | |  | S | | S |  | |  |  | |  | N | Y | | T |  | |  |  |
| H3N2 Virus Strain | | | | | | **Amino acid position in NA protein** | | | | | | | | | | | | | | | | | | | | | | | | | | | | | | | | | | | | | | | | |  |  |  |
|  |  |  |  |  |  | 58 | | | 65 | | | 79 | | | 150 | | | 197 | | | 221 | | | | 245 | | | 247 | | | 267 | | | 339 | | | 380 | | | 392 | | | | 468 | | |  |  |  |
| A/Texas/50/2012^b^ | | | | | | I | | | I | | | P | | | H | | | D | | | E | | | | S | | | S | | | T | | | D | | | I | | | I | | | | P | | |  |  |  |
| A/GNVL/01/2014^C^ | | | | | | M | | |  | | |  | | | R | | |  | | |  | | | |  | | |  | | |  | | | G | | |  | | |  | | | |  | | |  |  |  |
| A/GNVL/05/2014^d^ | | | | | |  | | | V | | | S | | | R | | |  | | | D | | | |  | | |  | | |  | | |  | | |  | | | T | | | |  | | |  |  |  |
| A/GNVL/06/2014^d^ | | | | | |  | | | V | | | S | | | R | | |  | | | D | | | |  | | |  | | |  | | |  | | |  | | | T | | | |  | | |  |  |  |
| A/GNVL/07/2014^d^ | | | | | |  | | | V | | | S | | | R | | |  | | | D | | | |  | | |  | | |  | | |  | | |  | | | T | | | |  | | |  |  |  |
| A/GNVL/08/2014^d^ | | | | | |  | | | V | | | S | | | R | | |  | | | D | | | |  | | |  | | |  | | |  | | |  | | | T | | | |  | | |  |  |  |
| A/GNVL/09/2014^e^ | | | | | |  | | | V | | | S | | | R | | |  | | | D | | | |  | | |  | | |  | | |  | | |  | | | T | | | |  | | |  |  |  |
| A/CH/9715293/2013^f^ | | | | | |  | | |  | | |  | | | R | | |  | | | D | | | |  | | |  | | |  | | |  | | |  | | | T | | | |  | | |  |  |  |
| A/GNVL/01/2016^g^ | | | | | |  | | |  | | |  | | | R | | | N | | | D | | | | N | | | T | | | K | | | N | | | V | | |  | | | | H | | |  |  |  |
| A/GNVL/02/2016^h^ | | | | | |  | | |  | | |  | | | R | | | N | | | D | | | | N | | | T | | | K | | | N | | | V | | |  | | | | H | | |  |  |  |
| A/ENVR/GNVL/01/2016 | | | | | |  | | |  | | |  | | | R | | | N | | | D | | | | N | | | T | | | K | | | N | | | V | | |  | | | | H | | |  |  |  |
| A/ENVR/GNVL/02/2016 | | | | | |  | | |  | | |  | | | R | | | N | | | D | | | | N | | | T | | | K | | | N | | | V | | |  | | | | H | | |  |  |  |
| A/ENVR/GNVL/03/2016 | | | | | |  | | |  | | |  | | | R | | | N | | | D | | | | N | | | T | | | K | | | N | | | V | | |  | | | | H | | |  |  |  |

^a^Yamashita A, Kawashita N, Kubota-Koketsu R, Inoue Y, Watanabe Y, Ibrahim MS, Ideno S, Yunoki M, Okuno Y, Takagi T, Yasunaga T, Ikuta K.2010. Highly conserved sequences for human neutralization epitope on hemagglutinin of influenza A viruses H3N2, H1N1, and H5N1: implication for human monoclonal antibody recognition. *Biochem Biophys Res Commun*. *393* (4) 614–618.

^b^Virus in 2013 – 2014 and 2014 – 2015 vaccines, Northern Hemisphere; GenBank accession number KC892952.1.

^c^GenBank Accession number KJ439217.

^d^Viruses in nasopharyngeal swabs collected in November 2014.

^e^Virus in sputum collected in November 2014.

^f^Virus (A/Switzerland/9715293/2013) in 2015 -2016 vaccine, Northern Hemisphere; EPI_ISL_165829.

^g^Viruses in nasopharyngeal swabs collected in March 2016 (identical sequences; A/GNVL/01/2016 deposited as GenBank # KX133410.1).

^h^NA amino acid sequence identical to that of A/GNVL/01/2016(H3N2).
